# Spring Rest-Grazing Time Influenced Soil Phosphorus Fractions by Altering the Abundance of Genes Involved in Phosphorus Cycling in a Subalpine Meadow

**DOI:** 10.3390/microorganisms13112618

**Published:** 2025-11-18

**Authors:** Hong Xiao, Yuanyuan Jing, Kai Ma, Yun Wang, Changlin Xu, Xiaojun Yu

**Affiliations:** 1 Grassland Ecosystem Key Laboratory of Ministry of Education, College of Pratacultural Science, Gansu Agricultural University, Lanzhou 730070, China; xiaoh@gsau.edu.cn (H.X.); jingyuanyuan@caas.cn (Y.J.); 1073325020104@st.gsau.edu.cn (K.M.); 1073325020101@st.gsau.edu.cn (Y.W.); xucl@gsau.edu.cn (C.X.); 2 Grassland Research Institute, Chinese Academy of Agricultural Sciences, Hohhot 010010, China

**Keywords:** phosphorus fractions, phosphorus cycling functional genes, subalpine meadow, rest-grazing

## Abstract

Soil phosphorus (P) availability is a critical factor limiting plant growth and ecosystem productivity that can be strongly influenced by land use factors, such as grazing by livestock. Seasonal grazing management can benefit grassland productivity and soil nutrient cycling in alpine meadows, but its effects on soil P availability and the microbial processes driving P transformation remain poorly understood. To address this, a long-term field experiment was conducted with five different spring rest-grazing periods, where soil P fractions were examined and metagenomic sequencing was employed to assess the functional profiles of microbial genes involved in P cycling. Early spring rest-grazing led to higher concentrations of labile P fractions (Resin-P and NaHCO_3_-Pi), indicating improved soil P availability. Moreover, rest-grazing in early spring significantly reduced HCl-Pi concentration while increased the concentration of conc. HCl-Po. Metagenomic analysis revealed that early spring rest-grazing may have contributed to a higher relative abundance of the organic P mineralization gene *phnA* but decreasing the relative abundance of inorganic P solubilization genes *ppa*, and P-uptake and transport gene *pstB*. The dominant microbial genera involved in P cycling were *Rhodopseudomonas* and *Mesorhizobium*. Soil temperature and water infiltration rate, both affected by early rest-grazing, were identified as the main environmental variables correlated with P-cycling functional gene composition. These influenced taxa with functional genes involving organic P mineralization, inorganic P solubilization, and P-uptake and transport, which may associate with enhancing soil labile P. This study provides insights into potential microbial processes under grazing management in grassland ecosystems.

## 1. Introduction

The phosphorus (P) cycle is a critical component of terrestrial ecosystems, influencing plant productivity, nutrient dynamics, and overall ecosystem health [1,2]. However, much of P in soil is bound in organic matter or in insoluble forms that are not readily available to plants [3]. Understanding the P cycle and how it is influenced by different land use is important for ensuring sustainable land use and maintaining ecosystem services [4]. For example, livestock grazing is one of the most widespread uses of grasslands that can strongly disrupt inorganic P (Pi) and organic P (Po) fractions [5,6,7]. However, the mechanisms underlying the impacts of grazing practices on soil P availability and pools are still not well understood; the understanding of this would greatly improve grassland management practices.

Soil microorganisms are central to the dynamics of the P cycle and the transition between P fractions. These microbial processes include the solubilization of Pi, mineralization of Po, uptake and transport of P, and the regulation of microbial responses to P deficiency [8,9]. The process of Pi solubilization converts the unavailable P into bioavailable forms, enhancing plant access to this essential nutrient [10]. For example, the expression genes (i.e., *gltA* and *pqqC*) involved in the production of organic acids facilitate the transformation of inorganic P, making it an essential mechanism in soils with high P fixation [11]. The decomposition of Po into Pi forms is crucial, especially in soils with a high content of organic matter, as it enhances P availability for plant uptake [12]. Specific microbial genes, including *aphA*, *phoN*, *phoA*, and *phoD*, are essential for the conversion of Po into usable forms [13,14]. Furthermore, microbial genes related to P transport such as *pit* and *pst* (phosphate transporters) facilitate the absorption and movement of P within microbial cells [15,16].

Livestock grazing is a key practice for the utilization of alpine grasslands [17], managed using grazing practices such as rotational and seasonal grazing, which can contribute to both environmental protection and economic benefits [18,19]. In seasonally grazed environments, spring grazing can damage grass regeneration and the ecological function of alpine grasslands [20]. One way to alleviate these effects is through rest-grazing in spring, which can minimize plant overfeeding during the regreening period, thus potentially enhancing grassland productivity [17,21]. Rest-grazing in early spring increased the utilization of available nutrients, soil bacterial diversity, and productivity in subalpine meadows on the Qinghai–Tibet Plateau [17,19,21,22]. Moreover, the activity of these P-cycling microorganisms is highly influenced by environmental factors such as soil temperature and moisture [11,23], each of which can be strongly influenced by anthropogenic land uses such as grazing [24,25]. However, whether different grazing periods induced the change in these environmental factors driving P cycling functional genes to influence soil P availability remains unclear.

To address the above issues, this study determined the content of different Pi and Po fractions and used metagenomics to detect P transformation and related microbial communities in soils treated with five different types of spring rest-grazing periods. It specifically hypothesizes that (1) Rest-grazing in early spring increases soil-labile P content compared to the traditional rest-grazing period in later spring; (2) Rest-grazing in early spring promotes the microbial processes of Pi solubilization and Po mineralization, resulting in the increase in soil labile P content; (3) Soil temperature and moisture are key factors driving microbial processes of P transformation in response to different spring rest-grazing periods. In this study, we focused on twenty-one representative genes consistently reported as core markers of microbial phosphorus cycling in soils based on previous studies [8,16]. These genes include *phnA*, *phnL/K*, *phoA*, and *phoD* involved in organic P mineralization, *ppa* and *ppx* related to inorganic P solubilization, and *ugp* and *pst* responsible for P uptake and transport. They were selected because they encode key enzymes and transporters directly mediating the conversion and mobilization of P in soil ecosystems, providing a robust basis for assessing microbial functional potential in P cycling.

## 2. Materials and Methods

### 2.1. Site Description and Experimental Design

The long-term rest-grazing experiment was established in January 2018 in an alpine meadow in the north-eastern Qinghai–Tibet Plateau, China (37°40′ N, 102°32′ E). The area is characterized by a distinctive plateau without an absolute frost-free period. The annual mean temperature is −0.1 °C (average annual accumulated temperature is 1380.0 °C) and the average annual precipitation is 416.9 mm, with three-quarters fall in the summer. The soil is an alpine chernozem, with 152.4 g·kg^−1^ organic matter, 6.6 g·kg^−1^ total nitrogen, and 0.8 g·kg^−1^ total phosphorus. Grazing typically occurs during November through the following year’s re-greening period. The established species of this area include *Carex capillifolia*, *Polygonum viviparum,* and *Elymus nutans* [19].

Five rest-grazing treatments were established based on soil thawing depth and plant re-greening, and the key dates for rest-grazing, plot conditions, livestock number, and plot area for each treatment are presented in Table 1. In each plot, one adult yak (Y) and one adult Tibetan sheep (S) were defined as a grazing unit.

### 2.2. Soil Sampling

Topsoil (0–10 cm) samples were collected from each plot using a 2 cm diameter soil corer on 2 June (the local traditional rest-grazing time) and 18 July (the vigorous grass growing season) in 2022. Within each grazing enclosure treatment, five representative sampling plots, each measuring 15 m by 15 m, were established to serve as replicates for the study. Within each plot, 6 soil cores were extracted following a Z-line. Soil samples from the same plot were homogenized to form a single composite sample. This sampling regimen was implemented in the fifth year of the rest-grazing experiment. Soil samples were passed through a 2 mm sieve to remove roots and organic debris and divided into three subsamples. One part was stored at −80 °C for DNA extraction; the other part was stored at 4 °C to measure soil-water content within one week; and the last part was air-dried to determine basic physicochemical properties and soil P fractions.

### 2.3. Determination of Soil Physicochemical Properties

Soil was lightly cut with a ring knife (100 cm^3^) to determine a bulk density (BD). Soil pH was determined in a 1:5 soil-water suspension using a pH meter (FE20-FiveEasyTM, Mettler Toledo, Gießen, Germany). Soil-water content (SWC) and soil temperature (ST) were measured using a soil moisture thermometer (TDR350, Spectrum, Middleton, WI, USA), and soil compactness (SD) was measured using a digital soil compaction tester (SC-900, Spectrum, Middleton, WI, USA). The soil infiltration rate (IR) is expressed as the average infiltration rate (mm min^−1^). Soil samples to determine BD, SD, IR, SWC, and ST were collected from positions adjacent to the soil cores of the Z-line, repeated 6 times per sample plot.

For soil physiochemical measurements, soil samples were first passed through a 0.15 mm sieve. Soil organic carbon (SOC) was determined using the K_2_Cr_2_O_7_ oxidation method. Total Nitrogen was analyzed using an elemental auto-analyzer (Vario MAX CN; Elementar, Hanau, Germany). Total Phosphorus was determined by digesting soil samples in a 10 mL of mixed acid solution (HNO_3_:HClO_4_:H_2_SO_4_ = 8:1:1, *v*/*v*/*v*) and measured at 660 nm with an ultraviolet-visible spectrophotometer (UV-1800, Shimadzu, Kyoto, Japan) after reaction with molybdenum blue. Dissolved organic C (DOC) was extracted with 0.5 M K_2_SO_4_ and quantified on a TOC analyzer (TOC-VCPH, Shimadzu, Japan). Soil NH_4_^+^-N and NO_3_^−^-N were measured using automated discrete analyzers (Smartchem 450, AMS, Pavia, Italy) in 0.05 M K_2_SO_4_ extracts.

### 2.4. Soil P Fractions Analysis

The fractions of soil P were analyzed using the modified Hedley phosphorus classification method [26]. Specifically, 0.5 g of air-dried soil (0.149 mm sieve) was placed in a 50 mL centrifuge tube with an anion-exchange resin membrane. Next, 30 mL of deionized water was added and then centrifuged for 10 min (4 °C, 10,000 r/min). The phosphorus fraction was extracted through a series of extractants to obtain the resin membrane (extract Resin-P), 0.5 M NaHCO_3_ (extract NaHCO_3_-P), 0.1 M NaOH (extract NaOH-P), 1 M HCl (extract HCl-Pi), hot concentrated HCl (extract conc. HCl-P), and 0.5 M H_2_SO_4_ (extract Residual-P). Each of the Pi fractions were colored using ammonium molybdate and ascorbic acid, and determined using automated fluorometric plate-reader (SpectraMax i3x, Molecular Devices, San Jose, CA, USA); the total fractions were determined via the colorimetric measurement after ammonium persulfate autoclaved at 121 °C. The concentrations of the Po fractions were calculated as the difference between the total fractions and the Pi fractions. A total of nine P fractions were determined (Resin-P, NaHCO_3_-Pi, NaHCO_3_-Po, NaOH-Pi, NaOH-Po, conc. HCl-Pi, conc. HCl-Po, HCl-Pi, and Residual-P), but the measurement of NaHCO_3_-Po was below the detectable limit in this study.

### 2.5. DNA Extraction, Library Construction, and Metagenomic Sequencing

To extract genomic DNA, we used OMEGA Soil DNA Kit (D5625–01, Omega Bio-tek Inc., Norcross, GA, USA) following the manufacturer’s instructions; DNA integrity was checked by electrophoresis on 1% agarose gels, while DNA concentration and purity were determined using NanoDrop spectrophotometer (Thermo Fisher Scientifc, Weihao, Waltham, MA, USA). Constructing libraries of the extracted DNA and paired-end sequencing was performed by Wekemo Tech Group Co., Ltd. (Shenzhen, China). The raw sequencing reads were generated by Illumina NovaSeq 6000 platform (Illumina, Inc., San Diego, CA, USA). Clean data were then obtained by quality control of raw sequencing reads with Trimmomatic [27] and Bowtie 2 [28]. The clean data were taxonomically categorized using Kraken 2 [29]. For function analysis, data were aligned with the protein database UniRef90 using HUMAnN3 software (Version 3.1) based on DIAMOND [30]. HUMAnN3 default comparison parameters were set as follows: translated query coverage threshold = 90.0, prescreen threshold = 0.01, evalue threshold = 1.0, translated subject coverage threshold = 50.0. Reads that failed these comparisons were filtered out and the relative abundance of proteins in UniRef90 was counted. To detect the taxonomic composition and abundance functional genes associated with soil P-cycling, genes associated with soil microbial P transformation were used. The 21 detected genes were classified into four categories (see detailed information in Table S1).

### 2.6. Statistical Analysis

Analysis of variance (ANOVA) was carried out using SPSS 22.0 software (Statistical Graphics Crop, Princeton, NJ, USA) to determine the effects of different spring rest-grazing periods on soil physicochemical properties, P fractions, and P-cycling functional genes. Several measures of diversity of the P-cycling microbial communities were estimated, including the Chao extrapolation of species richness, as well as the Shannon and Simpson diversity indices. These were calculated using the Majorbio Cloud Platform (https://cloud.majorbio.com/page/tools/, accessed on 24 October 2024). To analyze relationships between the soil physicochemical properties and P-cycling microbial community composition, redundancy analysis (RDA) and Pearson correlation analysis were employed. To analyze the association of soil phosphorus fractions with P-cycling functional genes, a Mantel test was calculated using *linkET* package in R (Version 4.2.2). To analyze the relative importance of P-cycling functional genes involved in organic P mineralization and inorganic P solubilization to soil-labile P and moderately labile P, random forest analysis was employed using the *randomForest* package in R.

## 3. Results

### 3.1. Soil Physicochemical Properties

Results from this study on the effects of different rest-grazing periods on soil physicochemical properties are presented in Table S2. Several variables, including ST, SWC, IR, SD, TP, NH_4_^+^-N, NO_3_^−^-N, SOC, and DOC, were significantly influenced by different rest-grazing treatments. The levels of ST, IR, and SD increased when rest-grazing time was postponed (from ST1 to CK), while SWC decreased. Compared with the traditional rest-grazing period (CK), rest-grazing in early spring, especially in ST1, increased the accumulation of SOC and decreased the accumulation of NH_4_^+^-N. The response of NO_3_^−^-N and DOC to rest-grazing treatments varied with sampling dates. Specifically, rest-grazing in early spring (ST1 and ST2) decreased the accumulation of NO_3_^−^-N and DOC in June, whereas ST1 and ST2 recorded the highest value of DOC and NO_3_^−^-N in July, respectively. The levels of BD, TN, and pH did not vary among rest-grazing treatments.

**Figure 1:**
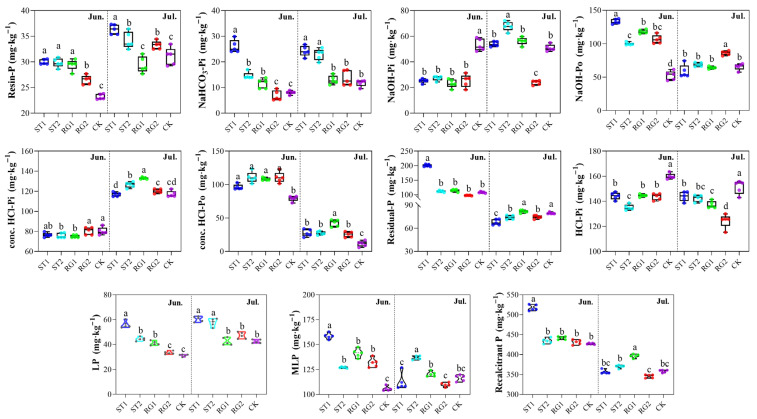
Effects of different rest-grazing periods on soil inorganic (Pi) and organic P (Po) fractions. LP (Labile-P) = Resin-P + NaHCO_3_-Pi; MLP (Moderately labile-P) = NaOH-Pi + NaOH-Po; Recalcitrant-P = conc. HCl-Pi + conc. HCl-Po + Residual-P + HCl-Pi. Different lowercase letters in the figure indicate significant differences between treatments (*p* < 0.05). Topsoil samples were collected on 2 June (Jun.) and 18 July (Jul.) in 2022, respectively. ST1, ST2, RG1, RG2, and CK indicates different rest-grazing treatments, including soil surface which began to thaw, soil thawing depth was more than 10 cm, re-greening coverage reached 30–40%, re-greening coverage reached 80%, dominant plant height ~5 cm, respectively.

When rest-grazing time was postponed, TP was reduced on both sampling dates (Table S2). For labile P fractions, the concentrations of Resin-P and NaHCO_3_-Pi increased when the rest-grazing time was earlier on both sampling dates (Figure 1). For moderately labile P fractions, the concentration of NaOH-Pi was significantly reduced, whereas the concentration of NaOH-Po was higher when the rest-grazing time was earlier in June. In July, the concentrations of NaOH-Pi and NaOH-Po only differed from the CK treatment in RG2. The total concentration of recalcitrant P was higher than both moderately labile P and labile P concentrations. Compared with CK, rest-grazing in early spring led to higher concentrations of conc. HCl-Po, while the concentration of HCl-Pi was lower.

### 3.2. Composition and Diversity of the P-Cycling Microbial Community

**Figure 2:**
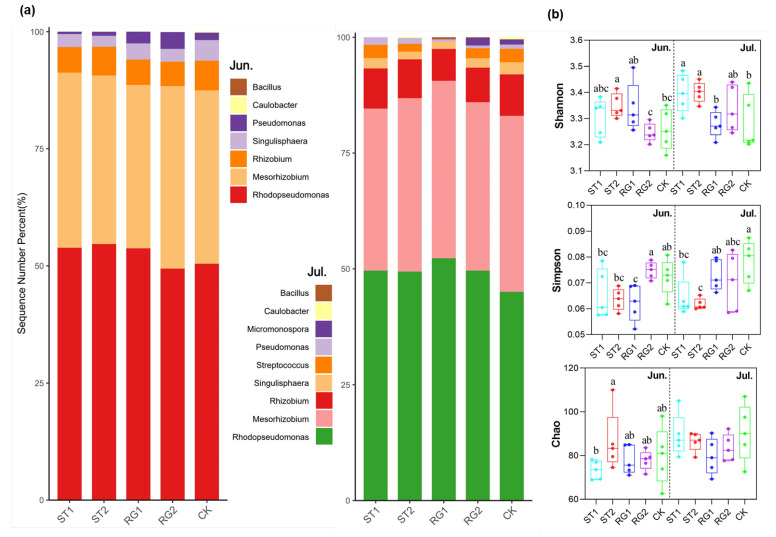
Composition and diversity of soil P-cycling microbial community in response to different rest-grazing periods. (**a**) Taxonomic composition of soil P-cycling microbial community at the genus level (unclassified taxa not shown). (**b**) Species richness (Chao), Simpson and Shannon diversity indices of at the species level. Different lowercase letters indicate significant differences between treatments (*p* < 0.05). Topsoil samples were collected on 2 June (Jun.) and 18 July (Jul.) in 2022, respectively. ST1, ST2, RG1, RG2, and CK indicates different rest-grazing treatments, including soil surface which began to thaw, soil thawing depth was more than 10 cm, re-greening coverage reached 30–40%, re-greening coverage reached 80%, dominant plant height ~5 cm, respectively.

The relative abundance of two genera, *Rhodopseudomonas* and *Mesorhizobium*, accounted 80% of the classified taxa (Figure 2a). The relative abundance of *Rhodopseudomonas* was higher with several rest-grazing treatments (ST1, ST2, and RG1) compared to that of traditional rest-grazing period on both sampling dates. However, diversity differed with the traditional rest-grazing period when compared to rest-grazing in early spring (ST1 and ST2). While there was no difference in the measured species richness, the Shannon index was higher, while the Simpson index was lower compared to traditional rest-grazing period (Figure 2b).

### 3.3. Abundance of P-Cycling Functional Genes

**Figure 3:**
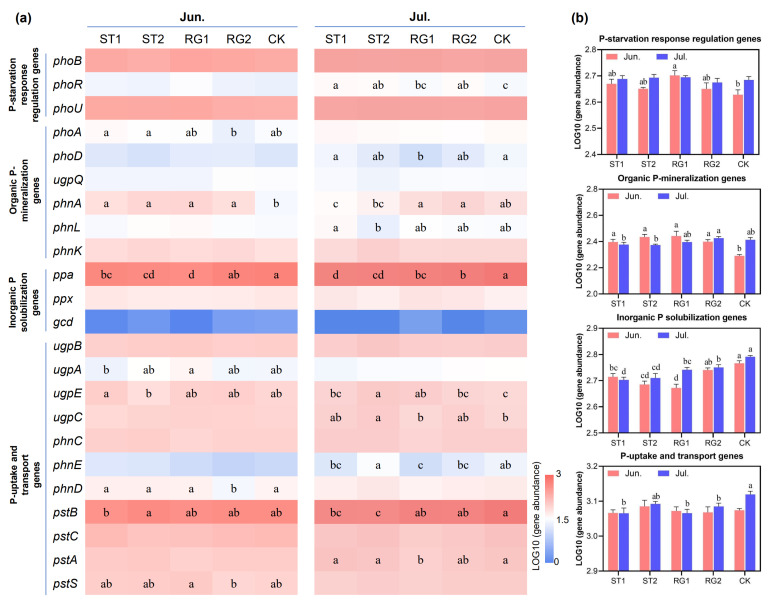
Abundances of soil P-cycling functional genes in response to different rest-grazing periods. (**a**) The heat map presents the log10-transformed abundances of 21 genes related to soil P-cycling. (**b**) The bar plot shows the log10-transformed total abundances of functional genes related to P-starvation, organic P-mineralization, inorganic P solubilization, and P-uptake and transport. Different lowercase letters indicate significant differences between treatments (*p* < 0.05). Topsoil samples were collected on 2 June (Jun.) and 18 July (Jul.) in 2022, respectively. ST1, ST2, RG1, RG2, and CK indicate different rest-grazing treatments, including soil surface which began to thaw, soil thawing depth was more than 10 cm, re-greening coverage reached 30–40%, re-greening coverage reached 80%, dominant plant height ~5 cm, respectively.

According to functional analysis of sequence data, a total of 21 genes related to soil P-cycling were identified; these were classified into four functional groups, including P-starvation response regulation, organic P-mineralization, inorganic P solubilization, and P-uptake and transport (Figure 3). The relatively higher abundance of organic P-mineralization genes was observed under rest-grazing in early spring (ST1 and ST2) in June but was reduced in July (Figure 3b). Among organic P-mineralization groups, the relative abundance of the *phnA* gene was affected by different rest-grazing periods (Figure 3a). The relative abundance of *ppa* and *pstB* genes were substantially higher than that of other genes, which dominated the collective abundance of inorganic P solubilization, and P-uptake and transport groups, respectively (Figure 3a). The collective abundance of inorganic P solubilization genes was relatively higher when the rest-grazing time was postponed (from ST1 to CK) on both sampling dates. The collective abundance of P-uptake and transport genes was relatively lower under rest-grazing in early spring (ST1, RG1, and RG2) compared with CK in July (Figure 3b).

### 3.4. Relationships Between Soil Physicochemical Properties and P-Cycling Functional Genes

**Figure 4:**
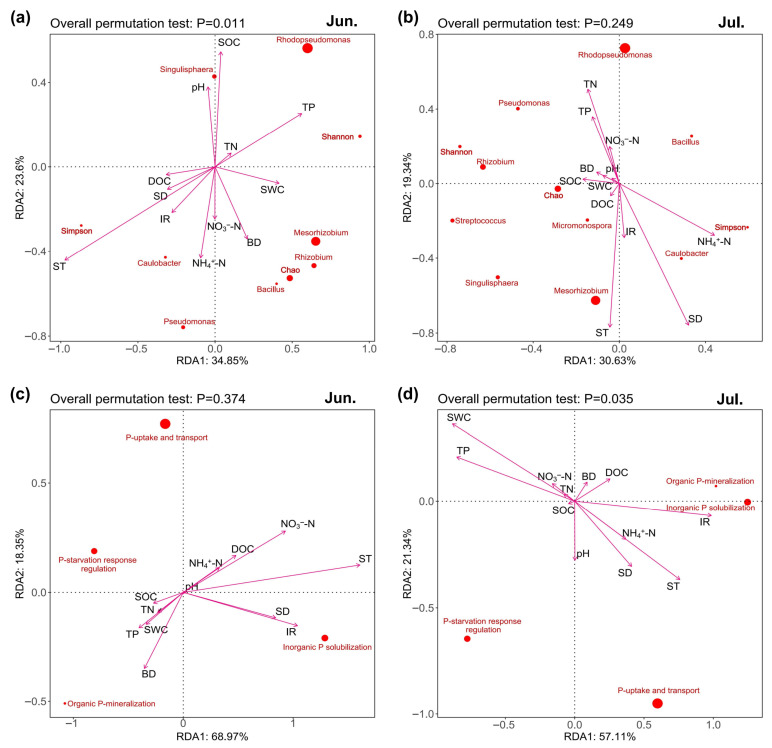
Redundancy analysis on the relationships among soil physicochemical properties, P-cycling microbial community composition and diversity, and P-cycling functional gene abundance in June and July. (**a**,**b**), the relationships between soil physicochemical properties and P-cycling microbial community composition in June and July, respectively. (**c**,**d**), the relationships between soil physicochemical properties and P-cycling functional gene abundance in June and July, respectively.

RDA analysis also showed that ST and IR were important environmental factors for soil P-cycling microbial community functional genes (Figure 4a–d). ST and IR were positively correlated with the collective abundances of inorganic P solubilization genes on both sampling dates (Figure 4c,d and Figure S1b), and positively correlated with the Simpson index, but negatively correlated with the abundance of *Rhodopseudomonas* in June (Figure 4a and Figure S1a). Moreover, the Simpson index positively correlated with the collective abundances of inorganic P solubilization genes on both sampling dates (Figure S1c). The abundance of *Rhodopseudomonas* positively correlated with P-starvation response regulation, organic P-mineralization, and inorganic P solubilization genes in June (Figure S1c).

### 3.5. Relationships Between Soil P Fractions and P-Cycling Functional Genes

**Figure 5:**
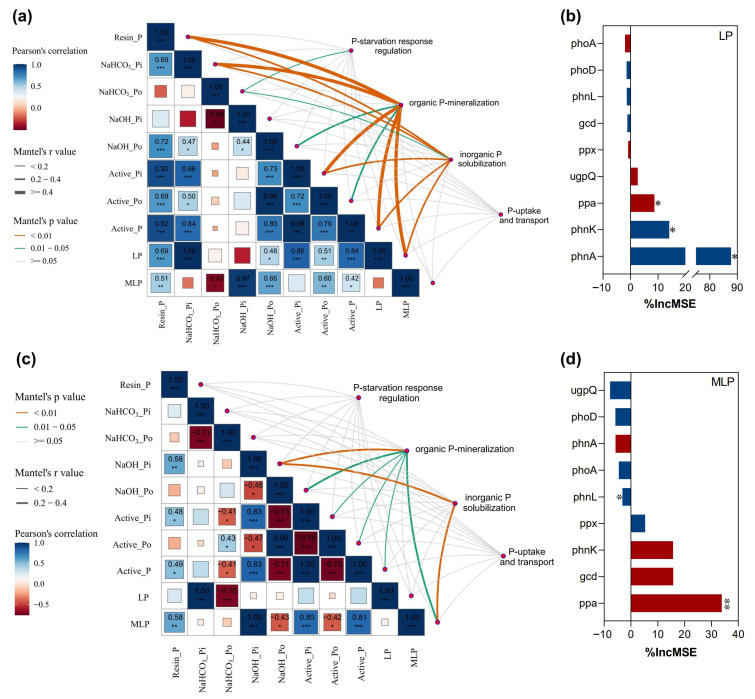
The relationship between soil P fractions and functional gene abundances. (**a**,**c**), Mantel test of the correlations between the P-cycling functional genes involved in P-uptake and transport, organic P-mineralization, inorganic P solubilization, and P-starvation response regulation and soil P fractions in June and July. (**b**,**d**), Contribution of soil P-cycling functional genes involved in organic P-mineralization and inorganic P solubilization to LP (Labile-P) based on the random forest model. %IncMSE, increase in MSE (%). *R*^2^ indicates the positive interpretation of soil P-cycling functional genes involved in organic P-mineralization and inorganic P solubilization to LP. Red denotes a negative correlation, whereas blue denotes a positive correlation. *, *p* < 0.05; **, *p* < 0.01; ***, *p* < 0.001.

Genes involved in organic P mineralization and inorganic P solubilization were correlated with LP, Resin-P, NaOH-Pi, NaOH-Po, conc. HCl-Po, and HCl-Pi in June (Figure 5a). Moreover, Organic P-mineralization genes had stronger correlations with NaOH-Po and conc. HCl-Po. In July, genes involved in organic P mineralization, inorganic P solubilization, and P-uptake and transport were correlated with labile P and NaHCO_3_-Pi (Figure 5c). The relative contributions of organic P-mineralization and inorganic P solubilization genes to labile P were further detected in the random forest model. The organic P-mineralization gene (*phnA*) and the inorganic P solubilization gene (*ppa*) were the primary indicators of labile P on both sampling dates (Figure 5b,d).

## 4. Discussion

### 4.1. Early Rest-Grazing Increased Soil P Availability

Varying rest-grazing periods significantly influence both labile and recalcitrant P fractions (Figure 1). These findings are consistent with recent research highlighting how grazing management alters nutrient cycling, including P dynamics, in grassland ecosystems [6,31,32]. Among the P fractions, the total concentration of labile P was both lower than moderately labile P and recalcitrant P concentrations (Figure 1), indicating relatively low soil P availability in the study region. Available P in livestock feces is higher than that found in the soil, thereby enhancing microbial processes involved in P mineralization and increasing the labile P in the soil [33,34]. Livestock trampling can also break up litter and accelerate the release of labile P levels in the soil [7,35]. However, continuous year-round grazing increases P export from the soil through forage and livestock products and decreases the P return to the soil by reducing the amount of litter, depleting the soil P pool [6,36]. Here, postponing the rest-grazing time led to lower Resin-P and NaHCO_3_-Pi (Figure 1), which are immediately available P fractions important for meeting the short-term P requirements [37]. This suggests that shortening the grazing period enhanced soil labile P concentrations available for plant regeneration and growth. These findings are in consistent with our first hypothesis.

Furthermore, early rest-grazing significantly influenced the fractions of organic P. Organic P serves as a long-term nutrient reservoir, gradually releasing P into the soil through microbial decomposition, thereby sustaining long-term fertility [38,39]. The observed shift toward greater organic P accumulation in the early rest-grazing treatments suggests that shorter grazing periods may facilitate more stable P storage and thus higher ecosystem productivity [40]. Additionally, the reduction in recalcitrant inorganic P (HCl-Pi) under early rest-grazing indicates that this management approach promotes the conversion of inorganic P into more biologically active organic forms, thereby supporting sustainable nutrient cycling and buffer against P depletion in grazed ecosystems [41,42].

### 4.2. Correlation of Soil P Availability and P-Cycling Functional Microbes

Understanding the functional genes involved in P cycling processes is helpful to improving soil P availability [43]. We found that the inferred abundances of organic P-mineralization and inorganic P solubilization genes correlated with labile-P concentrations, indicating that the potential microbial capacity for P transformation may contribute to the change in soil P availability [11,16]. Soil organic P mineralization genes play a key role in converting organic forms of phosphorus into inorganic forms that are usable [13,44]. Among organic P mineralization genes, the *phnA* gene encodes an enzyme that is involved in the initial steps of phosphonate degradation [45]. Indeed, the *phnA* gene abundance was the primary indicator of labile P in June in this study, suggesting that earlier rest-grazing treatments may have contributed to a relatively higher abundance of this gene, potentially leading to the increase in soil labile Pi. In addition, our finding suggests that the earlier rest-grazing treatment may regulate the relative abundance of the organic P mineralization gene to obtain more labile P when the NaOH-Po concentration was high, partially in accordance with our second hypothesis.

The fraction of NaOH-Pi can be solubilized by organic acids, such as gluconic acid, which are produced by the expression of inorganic P solubilization genes [46,47]. Enhancing the expression of inorganic P solubilization genes might be one of the reasons for the decrease in NaOH-Pi and the increase in labile P [11], potentially indicated by the abundance of inorganic P solubilization gene *ppa* in this study P (Figure 5b,d). These findings suggest that soil labile P may be associated with a relative higher abundance of the *ppa* gene to solubilize NaOH-Pi under traditional rest-grazing period. Conversely, a decrease in NaHCO_3_-Pi was also observed in the traditional rest-grazing period. This could be attributed to root uptake of available P and enhanced-microbial immobilization [8,48]. In addition, the *pstB* gene, which is part of the phosphate-specific transport (*Pst*) system plays a crucial role in phosphorus uptake [49,50], especially with low phosphate availability [51]. These results are consistent with those of Liu et al. [31], who demonstrated that the abundance of P-cycling related genes increased with grazing intensity, driving the transformation of stable P to labile P.

Some microbial taxa are linked to genes responsible for phosphorus solubilization and mineralization, such as *Proteobacteria* and *Actinobacteria* [51,52,53]. However, their contribution to P functional genes is not always proportional [11,16]. In this study, *Rhodopseudomonas* and *Mesorhizobium* dominated the community (~80% of the classified genera) (Figure 2), playing an important role in organic P mineralization and phosphate solubilization that enhance P availability [54,55,56,57]. Specifically, *Rhodopseudomonas palustris* was most the important *phnA* and *pstB*-harboring microbial species, while *Mesorhizobium loti* was the predominant species with the *ppa* gene (Figure S2). This suggests that *Rhodopseudomonas* and *Mesorhizobium* play a key role in driving soil P cycling in the present grassland ecosystem.

It is important to acknowledge that the functional gene analysis in this study was based on metagenomic KEGG Orthology (KO) annotations, which reflect the potential functional capacity of the microbial community rather than actual gene expression or enzymatic activity. Consequently, the observed differences in the abundance of P-cycling genes should be interpreted as indicative of the community’s genomic potential for phosphorus transformation, not as evidence of realized metabolic processes. Future studies integrating metatranscriptomic, proteomic, or enzyme activity assays are needed to confirm the transcriptional and functional responses of P-cycling microorganisms under different grazing regimes.

### 4.3. Factors Driving Soil P-Cycling Functional Microbes Responding to Rest-Grazing

Previous studies in this system revealed that rest-grazing in early spring increased the abundance and diversity of soil bacteria [19]. Here, rest-grazing in early spring also may have contributed to soil P-cycling microbial alpha diversity, with a higher Shannon index. A positive relationship between soil moisture and microbial diversity has been well documented [58,59,60]. However, the abundance of microbial P-cycling genes can be promoted under drought conditions [16]. With grazing, soil moisture levels tend to decrease, as shown here when the rest-grazing period was extended (Table S2), possibly due to a reduction in plant cover and consequent reduction in the biomass and activity of soil microbes [19,61,62]. The negative correlation between water content in the soil and the abundance of organic P mineralization and inorganic P solubilization genes (Figure S1b) is consistent with previous work by Gao et al. [16].

In addition to influencing soil moisture, the reduction in plant cover induced by grazing generally leads to an increase in soil temperature [24], which directly controls nutrient mineralization and absorption by regulating soil microbial activity [63]. Indeed, a recent study showed that the richness of the P cycling microbial community on the cold Tibetan plateau were more sensitive to warming than precipitation [64]. The microbial groups involved in phosphatase production and the mineralization of organic phosphorus that influences P cycling often increases with moderate warming [11,64,65]. For instance, phosphatase-encoding genes that mineralize organic phosphorus are often upregulated with warming [64]. Shi et al. [11] demonstrated that the decrease in gene abundance of inorganic P solubilization might be one of the reasons for the lack of solubilization of NaOH-Pi to Labile Pi, resulting in a decrease in soil labile Pi content at lower temperatures. Here, a positive relationship between temperature and the abundance of inorganic P solubilization genes suggests that increasing grazing time allows livestock to reduce plant biomass, increase temperature, and promote inorganic P solubilization to increase labile P for meeting the needs of plant growth and regeneration. These findings are in consistent with our third hypothesis.

By postponing the rest-grazing time, this study showed higher levels of soil-water infiltration, counter-acting the usual soil compaction that occurs through trampling [66]. However, more moderate grazing can promote soil water infiltration through improved root growth and organic matter input [67,68]. Postponing rest-grazing time induced an increase in lateral rooting extent and root length density, resulting in root accumulation in topsoil [21]. This can contribute to the increase in soil-water infiltration rate under the traditional rest-grazing period, which can enhance microbial processes involved in phosphorus cycling [59]. This is because microbial communities responsible for solubilizing phosphorus require adequate water flow to transport these agents and facilitate interactions between microbes and P-containing minerals [9].

## 5. Conclusions

This study demonstrates that early spring rest-grazing markedly enhanced soil phosphorus availability by increasing labile P fractions and altering the potential functional composition of microbial communities involved in P cycling. Earlier rest-grazing was associated with higher predicted abundances of genes related to organic P mineralization (*phnA*) and inorganic P solubilization (*ppa*), as well as changes in dominant microbial genera (*Rhodopseudomonas* and *Mesorhizobium*) that may contribute to phosphorus transformation. Soil temperature and water infiltration rate emerged as major environmental drivers shaping these putative microbial functional capacities. Our findings highlight that the timing of spring rest-grazing can influence soil physicochemical conditions and the potential microbial functions regulating P cycling, thereby providing valuable guidance for sustainable grazing management in subalpine meadows. It should be noted, however, that the functional profiles described here are predictive in nature and reflect potential rather than confirmed gene expression or enzyme activity, underscoring the need for future metatranscriptomic and biochemical validation.

## Figures and Tables

**Table 1:** Test design and plot condition.

Treatments	ST1	ST2	RG1	RG2	CK
Grazing date	1 March	1 March	1 March	1 March	1 March
Rest-grazing date	18 March	1 April	15 April	1 May	20 May
Plot conditions	Soil surface began to thaw	Soil thawing depth was more than 10 cm	Re-greening coverage reached 30–40%	Re-greening coverage reached 80%	Dominant plant height ~5 cm
Livestock number	4 (Y + S)	4 (Y + S)	4 (Y + S)	4 (Y + S)	16 (Y + S)
Plot area (m^2^)	1881	3344	4807	6478	33,855

Note: The daily requirement of adult yaks is 5.8 kg of hay, and the supplementary forage (oat) is 1.23 kg of hay. The daily requirement of adult Tibetan sheep is 1.7 kg of hay, and the supplementary forage (oat) is 0.22 kg of hay. The forage utilization rate was 80%. The grass yield was 2895 kg·hm^−2^ before the enclosure.

## Data Availability

The original contributions presented in the study are included in the article and Supplementary Material, further inquiries can be directed to the corresponding author.

## Supplementary Materials

The following supporting information can be downloaded at https://www.mdpi.com/article/10.3390/microorganisms13112618/s1, Table S1: The KO number, function descriptions, gene name, and classification of the investigated P cycling genes referring to KEGG database; Table S2: Effects of rest-grazing on soil physiochemical properties at different start times; Figure S1: Pearson’s correlation among soil physicochemical properties, P-cycling microbial community composition and diversity, and P-cycling functional gene abundances; Figure S2: Composition of *phnA*-, *ppa*-, *pstB*-harboring microbial species.
