# Supplementary material for: Spring Rest-Grazing Time Influenced Soil Phosphorus Fractions by Altering the Abundance of Genes Involved in Phosphorus Cycling in a Subalpine Meadow
Source: Microorganisms. 2025 Nov 18;13(11):2618. doi: 10.3390/microorganisms13112618 (PMC12654276; doi:10.3390/microorganisms13112618)
Supplement: Supplementary file 1 [file microorganisms-13-02618-s001.zip › microorganisms-3890440-supplementary.pdf]

Table S1 The KO number, function descriptions, gene name and classification of the investigated P cycling genes referring to KEGG database.

Classification	KO number	Details for gene function	Corresponding gene
Genes involved in P-starvation response regulation	K02039	*phoR/phoB* inhibitor protein *phoU*	*phoU*
	K07636	two-component system, OmpR family, phosphate regulon sensor histidine kinase *phoR*	*phoR*
	K07657	two-component system, OmpR family, phosphate regulon response regulator *phoB*	*phoB*
Genes involved in P-uptake and transport system	K05813	Glycerol-3-phosphate transporter subunit, periplasmic-binding component	*ugpB*
	K05814	Glycerol-3-phosphate transporter subunit	*ugpA*
	K05815	Glycerol-3-phosphate transporter subunit, membrane component	*ugpE*
	K05816	Glycerol-3-phosphate transporter subunit, ATP-binding component	*ugpC*
	K02041	phosphonate transport system, ATP-binding component	*phnC*
	K02042	phosphonate transport system, membrane component	*phnE*
	K02044	phosphonate transport system, periplasmic-binding component	*phnD*
	K02036	phosphate transport system, ATP-binding component	*pstB*
	K02037	phosphate transport system, membrane component	*pstC*
	K02038	phosphate transport system, membrane component	*pstA*
	K02040	phosphate transport system, periplasmic-binding component	*pstS*
	K03306	inorganic phosphate transporter	*pit*
Genes involved in organic P-mineralization	K06193	phosphonoacetate hydrolase	*phnA*
	K05780	C-P lyase subunit, alpha-D-ribose 1-methylphosphonate 5-triphosphate synthase	*phnL*
	K05781	Phosphonate transport system ATP-binding protein	*phnK*
	K01077	alkaline phosphatase	*phoA*
	K01113	alkaline phosphatase	*phoD*
	K09474	acid phosphatase (class A)	*phoN*
	K01126	Periplasmic glycerophosphoryl diester	*ugpQ*
Genes involved in inorganic P-solubilization	K00117	quinoprotein glucose dehydrogenase	*gcd*
	K01524	exopolyphosphatase / guanosine-5'-triphosphate,3'-diphosphate pyrophosphatase	*ppx*
	K01507	inorganic pyrophosphatase	*ppa*

Table S2 Effects of rest-grazing on soil physiochemical properties at different start times

Sampling date	Treatment	Soil physical properties					Soil chemical properties						
		ST (℃)	SWC (%)	BD (g/cm^3)	IR (mm/min)	SD (KPa)	TP (g/kg)	TN (g/kg)	NH_4^+-N (mg/kg)	NO_3^--N (mg/kg)	SOC (g/kg)	DOC (g/kg)	pH
Jun.	ST1	20.89±0.02c	25.30±0.38ab	0.64±0.03a	0.44±0.01b	1310.00±45.63b	0.85±0.03a	6.55±0.24a	22.04±1.32b	5.16±0.37c	96.84±0.84a	0.45±0.04b	7.81±0.01a
	ST2	20.26±0.02d	26.50±1.20a	0.67±0.02a	0.45±0.01b	1327.50±44.96b	0.82±0.02ab	6.87±0.07a	36.29±2.02a	6.23±0.24b	87.57±0.73b	0.43±0.05b	7.72±0.05a
	RG1	20.89±0.03c	23.39±0.71bc	0.69±0.03a	0.51±0.04b	1403.25±64.74b	0.80±0.01ab	6.63±0.11a	24.15±1.38b	5.14±0.20c	88.84±1.47b	0.56±0.06ab	7.71±0.06a
	RG2	22.32±0.04b	23.66±0.72b	0.67±0.01a	0.62±0.03a	1480.50±52.57b	0.79±0.02bc	6.33±0.45a	34.63±0.28a	5.60±0.13bc	86.54±0.50b	0.51±0.03ab	7.72±0.05a
	CK	22.81±0.03a	21.07±0.84c	0.64±0.02a	0.63±0.05a	1792.75±62.21a	0.73±0.02c	6.24±0.27a	34.48±0.56a	6.92±0.06a	86.10±0.32b	0.64±0.04a	7.78±0.03a
Jul.	ST1	21.31±0.08c	23.76±0.33a	0.76±0.02a	0.51±0.01b	1034.67±22.55b	0.86±0.03a	6.98±0.24a	15.63±0.21b	8.02±0.72b	85.70±0.74a	0.56±0.02a	7.79±0.02a
	ST2	21.50±0.23bc	23.19±0.31ab	0.75±0.01a	0.46±0.00b	1057.33±32.54b	0.83±0.01ab	6.50±0.21ab	15.86±0.33b	10.31±0.55a	78.18±0.65c	0.39±0.01b	7.76±0.02a
	RG1	21.40±0.29bc	22.99±0.21ab	0.67±0.00b	0.56±0.04b	1068.67±69.07b	0.83±0.02ab	6.50±0.14ab	21.09±0.30a	8.59±0.65ab	80.03±1.33bc	0.42±0.02b	7.70±0.04a
	RG2	22.08±0.25b	22.87±0.29b	0.76±0.02a	0.68±0.04a	1070.33±20.99b	0.78±0.02bc	6.26±0.09b	16.12±0.69b	8.23±0.74ab	78.67±0.46c	0.55±0.02b	7.75±0.02a
	CK	22.97±0.02a	22.60±0.17b	0.74±0.02a	0.73±0.04a	1319.50±63.72a	0.72±0.02c	6.39±0.27ab	20.90±0.13a	6.91±0.71b	82.00±0.31b	0.53±0.02a	7.78±0.02a

Note: Different lowercase letters in the table indicate significant differences between treatments ($p < 0.05$). ST: soil temperature, SWC: soil water content, BD: soil bulk density, IR: soil water infiltration rate, SD: soil compactness, TP: soil total phosphorus, TN: soil total nitrogen, SOC: soil organic carbon, DOC: dissolved organic carbon. Different lowercase letters in the table indicate significant differences between treatments ($P < 0.05$).

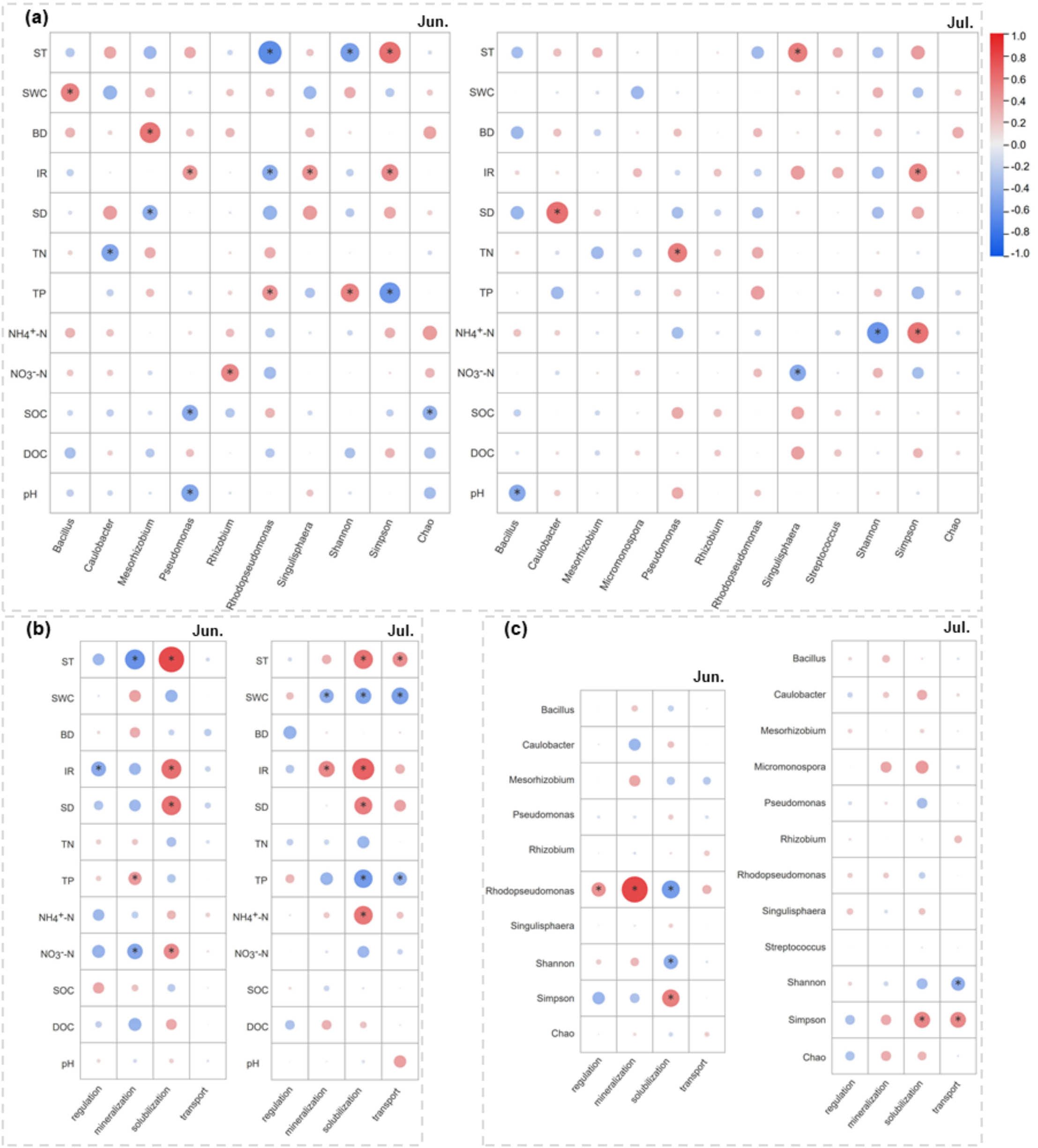

Figure S1 Pearson's correlation among soil physicochemical properties, P-cycling microbial community composition and diversity, and P-cycling functional gene abundances. *, $P < 0.05$; **, $P < 0.01$; ***, $P < 0.001$.

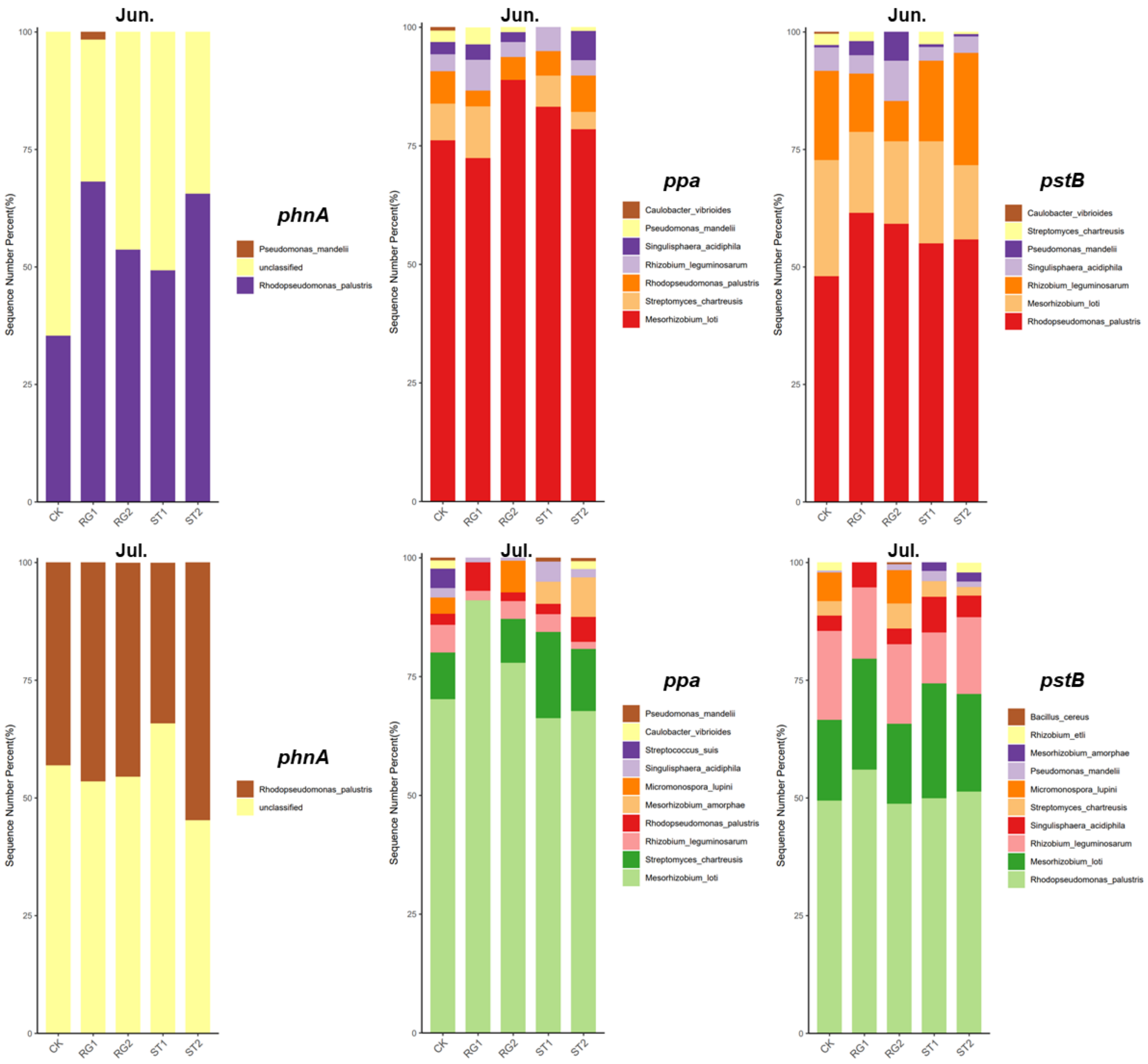

Figure S2 Composition of *phnA*-, *ppa*-, *pstB*-harboring microbial species
